# Weighted single-step GWAS and RNA sequencing reveals key candidate genes associated with physiological indicators of heat stress in Holstein cattle

**DOI:** 10.1186/s40104-022-00748-6

**Published:** 2022-08-20

**Authors:** Hanpeng Luo, Lirong Hu, Luiz F. Brito, Jinhuan Dou, Abdul Sammad, Yao Chang, Longgang Ma, Gang Guo, Lin Liu, Liwei Zhai, Qing Xu, Yachun Wang

**Affiliations:** 1 Laboratory of Animal Genetics, Breeding and Reproduction, Ministry of Agriculture of China, National Engineering Laboratory of Animal Breeding, College of Animal Science and Technology, China Agricultural University, No.2, Yuanmingyuanxi Road, Haidian District, Beijing, 100193 China; 2 Department of Animal Sciences, Purdue University, West Lafayette, IN 47907 USA; 3 College of Animal Science and Technology, Beijing University of Agriculture, Beijing, 100096 China; 4 College of Animal Science, Xinjiang Agricultural University, Urumqi, 830052 China; 5 Beijing Sunlon Livestock Development Company Limited, Beijing, 100029 China; 6 Beijing Dairy Cattle Center, Beijing, 100192 China; 7 College of Life Sciences and Bioengineering, Beijing Jiaotong University, Beijing, 100044 China

**Keywords:** Climatic resilience, Dairy cattle, QTL mapping, RNA sequencing, WssGWAS

## Abstract

**Background:**

The study of molecular processes regulating heat stress response in dairy cattle is paramount for developing mitigation strategies to improve heat tolerance and animal welfare. Therefore, we aimed to identify quantitative trait loci (QTL) regions associated with three physiological indicators of heat stress response in Holstein cattle, including rectal temperature (RT), respiration rate score (RS), and drooling score (DS). We estimated genetic parameters for all three traits. Subsequently, a weighted single-step genome-wide association study (WssGWAS) was performed based on 3200 genotypes, 151,486 phenotypic records, and 38,101 animals in the pedigree file. The candidate genes located within the identified QTL regions were further investigated through RNA sequencing (RNA-seq) analyses of blood samples for four cows collected in April (non-heat stress group) and four cows collected in July (heat stress group).

**Results:**

The heritability estimates for RT, RS, and DS were 0.06, 0.04, and 0.03, respectively. Fourteen, 19, and 20 genomic regions explained 2.94%, 3.74%, and 4.01% of the total additive genetic variance of RT, RS, and DS, respectively. Most of these genomic regions are located in the *Bos taurus* autosome (BTA) BTA3, BTA6, BTA8, BTA12, BTA14, BTA21, and BTA24. No genomic regions overlapped between the three indicators of heat stress, indicating the polygenic nature of heat tolerance and the complementary mechanisms involved in heat stress response. For the RNA-seq analyses, 2627 genes were significantly upregulated and 369 downregulated in the heat stress group in comparison to the control group. When integrating the WssGWAS, RNA-seq results, and existing literature, the key candidate genes associated with physiological indicators of heat stress in Holstein cattle are: *PMAIP1, SBK1, TMEM33, GATB, CHORDC1, RTN4IP1,* and *BTBD7*.

**Conclusions:**

Physiological indicators of heat stress are heritable and can be improved through direct selection. Fifty-three QTL regions associated with heat stress indicators confirm the polygenic nature and complex genetic determinism of heat tolerance in dairy cattle. The identified candidate genes will contribute for optimizing genomic evaluation models by assigning higher weights to genetic markers located in these regions as well as to the design of SNP panels containing polymorphisms located within these candidate genes.

**Graphical Abstract:**

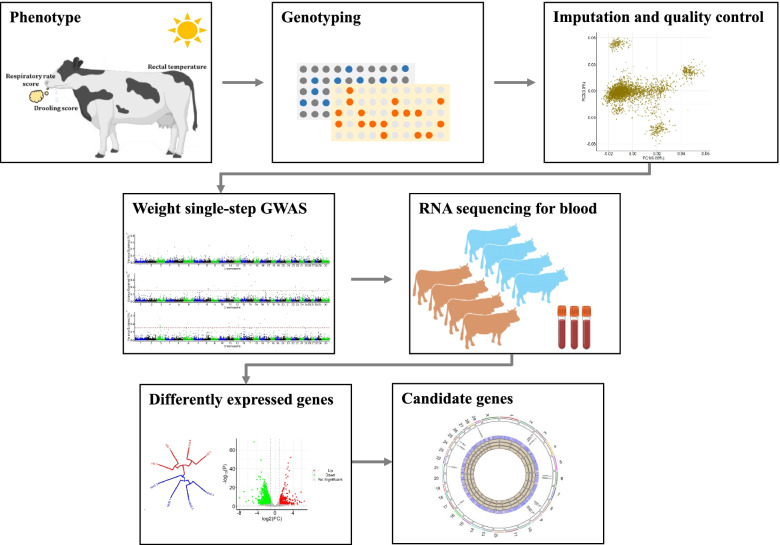

**Supplementary Information:**

The online version contains supplementary material available at 10.1186/s40104-022-00748-6.

## Introduction

A global warming trend has been observed over the last century [1], which has unfavorable effects on livestock production. Thus, heat stress is becoming a major challenge facing the global dairy industry [2]. Heat stress is the result of the sum of external forces acting on an animal that evokes a series of behavioral and physiological responses, including sweating, higher respiration rate, vasodilation with increased blood flow to skin surface, reduced metabolic rate, decreased dry matter intake, and altered water metabolism [3]. During periods of heat stress the hypothalamic-pituitary-adrenal (HPA) and the sympathetic-adrenal-medullary (SAM) axis are activated for maintaining homeostasis in response to stressful stimuli [4]. Under chronic stress, cortisol secretion associated with immune suppression [5, 6] leading to the animal becoming more susceptible to disease and immune challenges [7]. Heat stress is shown to severely alter the welfare, as well as productive and reproductive performance of dairy cows [8]. Due to the thermal hysteresis existing [9] for milk yield in dairy cattle, physiological performance traits are better indicators for monitoring heat load of individuals. We have established two scoring systems for respiration rate and salivation (drooling) in addition to measuring rectal temperature in Holstein population [10]. Respiration rate score (RR), rectal temperature (RT), and drooling score (DS) are lowly heritable traits controlled by numerous quantitative trait loci (QTL), each with a small effect, thereby making it more difficult to obtain fast genetic progress for these traits.

Genome-wide association studies (GWAS) are used to discover genomic regions associated with phenotypes of interest [11], and consequently, biological processes in which they are involved in. For instance, Luo et al. [12] identified candidate genes related to RT by using single-SNP regression GWAS. Subsequently, gene expression analyses were performed to validate the key functional genes identified [12]. A recent method known as single-step GWAS [13] is becoming the gold standard method for GWAS as it enables the simultaneous integration of genomic, phenotypic, and pedigree information in a single analysis. ssGWAS has been widely used for scanning QTLs associated with complex traits in dairy cattle such as milk yield and composition traits in Holstein cattle [14, 15]; fertility and reproductive disorders [16]; and heat tolerance [17]. Furthermore, the combination of GWAS and transcriptomics has been shown to significantly enhance the understanding of the genetic architecture of complex traits [18]. Understanding how genetic variation shapes the phenotypic variability of complex traits requires the identification of such genetic polymorphisms through GWAS combined with functional evaluation of the key candidate genes through RNA sequencing (RNA-seq). RNA-seq is a technique that evaluates the quantity and sequences of RNA in a sample based on next generation sequencing (NGS) tools. RNA-seq analyzes the full transcriptome, indicating which of the genes encoded in the DNA are turned on or off and to what extent [19]. To our best knowledge, no study has combined weighted single-step GWAS (WssGWAS) and RNA-seq for the determination of candidate genes and genetic variants associated with physiological indicators of heat stress in dairy cattle. Thus, the main objectives of this study were to: 1) identify genomic regions associated with RT, RR, and DS in Holstein cattle based on WssGWAS; 2) search for candidate genes in the QTL regions that explain greater proportions of the total additive genetic variance of each trait; and, 3) utilize RNA-seq and gene network analyses to investigate the biological processes shared by the candidate genes identified for the three indicators of heat tolerance.

## Material and methods

### Phenotype, pedigree and genotypic data

A total of 69,837 (RT), 40,760 (RR), and 40,889 (DS) phenotypic records were collected in 15,303 lactating Holstein cows from 2013 to 2020. The process of data collection, scoring protocol of RR and DS, and farm management have been described in details by Luo et al. [10]. The descriptive statistics for the phenotypic data and environmental index (temperature-humidity index, THI) are presented in Table 1. Each lactating cow was recorded twice a day for two consecutive days (07:00–11:00 and 14:00–18:00). A small proportion of cows were measured in more than one lactation (i.e., > 4 records/cow).

**Table 1:** Descriptive statistics of phenotypic records and environmental variables used in the analyses

Variable	NR	NC	Minimum	Maximum	Median	Mean	SD
Parity	74,041	15,304	1	10	2	2.19	1.37
Days in milk	74,041	15,304	1	921	188	194.8	126.31
Rectal temperature, °C	69,837	15,170	37.1	40.8	38.8	38.94	0.6
Respiration rate score	40,760	10,865	1	3	2	1.68	0.73
Drooling score	40,889	10,919	1	3	1	1.49	0.64
THI	74,041	15,304	70.5	90.2	81	80.75	3.67

*NR* number of records, *NC* number of cows, *SD* standard deviation, *THI* Temperature-humidity index

The pedigree file contained 38,101 animals, spanning over three generations. A total of 3200 animals (3119 cows and 81 bulls) were genotyped using the Illumina 150 K Bovine Bead chip (Illumina, Inc., San Diego, CA, USA), which contains 139,377 single nucleotide polymorphism (SNPs). The individuals with call rate higher than 0.9 were kept and the call rate of genotyped individuals is 0.977 before imputation. All of the genotyped animals were included in a genotype imputation analyses for imputing the missing SNPs. This was done using the Beagle5.1 software [20]. Genotype quality control kept SNPs with: minor allele frequency (MAF) greater than 0.05, no extreme departure from Hardy-Weinberg equilibrium (*P*-value greater than 10^−6^), known chromosome and genome position, and located in the autosomal chromosomes. After the quality control, 114,766 SNPs and 3200 animals were kept for further analyses.

### Statistical analyses

A multiple-trait model was employed to estimate variance and covariance components, which can be described as follows:

$$\boldsymbol{y}=\boldsymbol{Xb}+\boldsymbol{Za}+\boldsymbol{Wpe}+\boldsymbol{e}$$

where ***y*** is the vector of phenotypic records (RT, RR, and DS); ***b*** is the vector of systematic effects including farm-year (for RT) or farm-year-scoring person (for TT and DS), parity (1, 2, or 3+), lactation stage (days in milk 1–50, 51–100, 101–150, 151–200, 201–250, 251–300, or > 300), milking status (data collected before milking, after milking, or unknown), and THI (as a continuous covariable in the model fitting a linear regression); ***a*** is the vector of random additive genetic effects; ***p*** is the vector of random permanent environmental effects; ***e*** is the vector of random residuals; and ***X***, ***Z*,** and ***W*** are the incidence matrices linking phenotypic records to ***b*, *a*,** and ***pe*,** respectively. The model assumptions are:

$$\boldsymbol{a}\sim N\left(\mathbf{0},\boldsymbol{H}\otimes \left[\begin{array}{c}{\sigma}_{a(RT)}^2\ {\sigma}_{a\left( RT, RR\right)}\ {\sigma}_{a\left( RT, DS\right)}\\ {}{\sigma}_{a\left( RT, RR\right)}\ {\sigma}_{a(RR)}^2\ {\sigma}_{a\left( RR, DS\right)}\\ {}{\sigma}_{a\left( RT, DS\right)}\ {\sigma}_{a\left( RR, DS\right)}\ {\sigma}_{a(DS)}^2\ \end{array}\right]\right)$$

$$\boldsymbol{pe}\sim N\left(\mathbf{0},\boldsymbol{I}\otimes \left[\begin{array}{c}{\sigma}_{pe(RT)}^2\ {\sigma}_{pe\left( RT, RR\right)}\ {\sigma}_{pe\left( RT, DS\right)}\\ {}{\sigma}_{pe\left( RT, RR\right)}\ {\sigma}_{pe(RR)}^2\ {\sigma}_{pe\left( RR, DS\right)}\\ {}{\sigma}_{pe\left( RT, DS\right)}\ {\sigma}_{pe\left( RR, DS\right)}\ {\sigma}_{pe(DS)}^2\ \end{array}\right]\right)$$

$$\boldsymbol{e}\sim N\left(\mathbf{0},\boldsymbol{I}\otimes \left[\begin{array}{c}{\sigma}_{e(RT)}^2\ {\sigma}_{e\left( RT, RR\right)}\ {\sigma}_{e\left( RT, DS\right)}\\ {}{\sigma}_{e\left( RT, RR\right)}\ {\sigma}_{e(RR)}^2\ {\sigma}_{e\left( RR, DS\right)}\\ {}{\sigma}_{e\left( RT, DS\right)}\ {\sigma}_{e\left( RR, DS\right)}\ {\sigma}_{e(DS)}^2\ \end{array}\right]\right)$$

where ${\sigma}_{k\left(i,j\right)}^2$ is the variance of the effect k (**a**, **pe**, **e**) of trait *i*, *σ*_*k*(*i*, *j*)_ is the covariance between trait *i* and *j* for the effect *k*. ***H*** is the matrix that combines pedigree and genomic information [21], and ***I*** is an identity matrix. The inverse of ***H*** was calculated as [21]:

$${\boldsymbol{H}}^{-\mathbf{1}}={\boldsymbol{A}}^{-\mathbf{1}}+\left[\begin{array}{cc}\mathbf{0}& \mathbf{0}\\ {}\mathbf{0}& {\boldsymbol{G}}^{-\mathbf{1}}-{\boldsymbol{A}}_{\mathbf{22}}^{-\mathbf{1}}\end{array}\right]$$

where ***A*** is the numerator relationship matrix based on pedigree for all animals; ***A***_***22***_ is the numerator relationship matrix for genotyped animals. The ***G*** matrix was computed as [22]:

$$\boldsymbol{G}=\frac{\boldsymbol{Z}\boldsymbol{D}{\boldsymbol{Z}}^{\prime }}{\sum_{i=1}^M2{p}_i\left(1-{p}_i\right)}$$

where ***Z*** is a matrix of gene content adjusted for allele frequencies (0, 1, or 2 for aa, Aa, and AA, respectively); ***D*** is a diagonal matrix of weights for SNP variances (initially ***D*** = ***I***); ***M*** is the number of SNPs, and *p*_*i*_ is the minor allele frequency of the *i*^th^ SNP. Variance components were estimated using the average information-restricted maximum likelihood (AI-REML) procedure implemented in the AIREMLF90 package from the BLUPF90 family programs [23].

### Weighted single-step genome-wide association study (WssGWAS)

The estimates of SNP effects and weights for the WssGWAS analyses (three iterations) were obtained according to Wang et al. [24]. The weight for each SNP was calculated as: ${d}_i={1.125}^{\frac{\left|{\hat{a}}_i\right|}{sd\left({\hat{a}}_i\right)}-2}$[22], where *i* is the *i*^th^ SNP. The percentage of the total additive genetic variance explained by the *i*^th^ region was calculated as:

$$\frac{\mathrm{Var}\left(a_i\right)}{\upsigma_a^2}\times 100\%=\frac{\mathrm{Var}\left({\sum}_{j=1}^{10}{\mathrm{Z}}_j{\hat{\mathrm{u}}}_j\right)}{\upsigma_a^2}\times 100\%$$

Where *a*_*i*_ is genetic value of the *i*^th^ region that consists of contiguous 10 SNPs, ${\sigma}_a^2$ is the total additive genetic variance, ***Z***_***j***_ is a vector of gene content of the *j*^th^ SNP for all individuals, and ${\hat{u}}_j$ is the marker effect of the *j*^th^ SNP within the *i*^th^ region.

### Candidate genes detection and functional enrichment analyses

Genomic windows of 10 consecutive SNPs that explained 0.15% or more of the total additive genetic variance, based on the WssGWAS analyses, were considered to be associated with the studied traits. A Manhattan plot was created using the R software [25]. Genes were annotated on the basis of the starting and ending coordinates of each window (using the ARS-UCD1.2 assembly as the reference genome; GCA_002263795.2) by using the R package ‘Biomart’ of Ensembl [26]. Kyoto Encyclopedia of Genes and Genomes (KEGG) pathways and Gene Ontology (GO) terms were enriched via the “clusterProfiler” package [27].

### RNA sequencing

Eight primiparous cows with DIM ranging from 135 to 144 d (mid-lactation and pregnant) were selected from 3200 genotyped individuals for RNA-seq. Blood samples were collected via the coccygeal venipuncture in the Spring (4 cows in April during the thermoneutral period with average daily temperature lower than 25 °C) and Summer (4 cows in July during heat stress period with average daily temperature higher than 25 °C) seasons, following the RT, TT, and DS recording on the same day. RT, TT, and DS were collected after blood samples collected to reduce artificial stimulation. The experimental design in current study is consisted with other previous research investigating heat stress by RNA-seq [28, 29]. The RNA was isolated from blood according to the manufacturer’s instructions of the TRIzol Reagent method [30]. The RNA concentration and quality were assessed using the Equalbit RNA BR Assay Kit (Invitrogen, CA, USA) and Nanodrop 2000 (Thermo, Massachusetts, USA). RNA integrity was detected by 1% agarose gel electrophoresis and used for library construction with 28S/18S > 1. For RNA-seq library, 2 μg of RNA was used for purification and fragment using NEBNext Poly(A) mRNA Magnetic Isolation Module (Cat No. E7490S, New England Biolabs Ltd., Hitchin, Herts, UK) then followed by cDNA library with NEBNext Ultra RNA Library Prep Kit for Illumina (Cat No. E7530S, New England Biolabs Ltd., Hitchin, Herts, UK). All libraries were quantified by Equalbit DNA BR Assay Kit (Invitrogen, CA, USA) and pooled equimolarly. They were finally submitted for sequencing using the NovaSeq 6000 System (Illumina, Inc., San Diego, CA, USA) which generated 150 bases paired-end reads.

### Differential expression and functional analyses

The quality of the sequencing reads was evaluated using the FastQC software (v0.11.9) and global trimming using the Fastp [31]. All clean reads were mapped to the bovine genome of version ARS-UCD1.2 using the software STAR [32], and a Picard query [33] was carried out to eliminate duplicates. We investigated population structure through a principal component analysis (PCA) implemented in the PLINK software [34]. For differential expression gene screening, exact test based on quantile-adjusted conditional maximum likelihood (qCML) was performed using the edgeR [35] R package with criteria fold change ≥ 2 and 0.05 for the alpha of false discovery rate (4 samples vs. 4 samples). The heatmap was constructed using the pheatmap R package [36].

## Results

### Genetic parameter estimates

Estimates of variance components for RT, RR, and DS are shown in Table 2. The heritability estimates ranged from 0.03 (DS) to 0.06 (RT), with repeatability estimates ranging from 0.12 (DS) to 0.19 (RT), indicating the large environmental influence in these indicators of heat stress. Statistically significant genetic correlations were observed for RT with RR (0.22) and RR with DS (0.21).

**Table 2:** Genetic parameters, heritability, and repeatability for the evaluated physiological traits under heat stress

Trait	${\boldsymbol{\upsigma}}_{\mathbf{a}}^{\mathbf{2}}$	${\boldsymbol{\upsigma}}_{\mathbf{PE}}^{\mathbf{2}}$	${\boldsymbol{\upsigma}}_{\mathbf{e}}^{\mathbf{2}}$	Heritability (SE)	Repeatability
Rectal temperature	0.015	0.03	0.20	0.06(0.01)	0.19
Respiratory rate score	0.016	0.03	0.31	0.04(0.01)	0.13
Drooling score	0.011	0.03	0.29	0.03(0.01)	0.12

### QTL mapping and trait-related gene identification

**Fig. 1:**
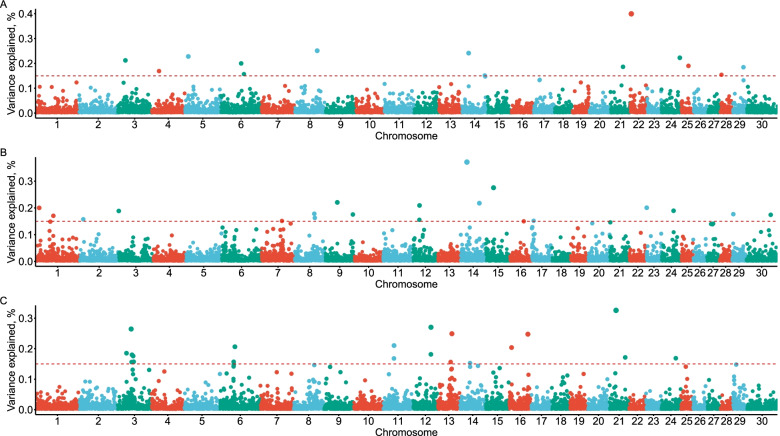
Proportion of the total additive genetic variance of 10-SNP genomic windows based on the weighted single-step genome association studies. **A** The Manhattan plot for rectal temperature. **B** The Manhattan plot for respiration rate score. **C** The Manhattan plot for drooling score. The red dashed lines represent the threshold 0.15% of the total additive genetic variance

Figure 1 presents the genetic variance of each genomic window after performing WssGWAS with three iterations. Fourteen, 19, and 20 genomic regions reached the pre-defined threshold (0.15%) and they explained 2.94%, 3.74%, and 4.01% of the total additive genetic variance for RT, RR, and DS, respectively. Most of these genomic regions are located in the *Bos taurus* autosome (BTA) BTA3, BTA6, BTA8, BTA12, BTA14, BTA21, and BTA24. However, we found no overlapping genomic regions between the three physiological traits in this study. The detailed information about these genomic regions related to the three physiological traits and candidate genes are presented in Table 3.

**Table 3:** Information of 10-SNP windows explaining more than 0.15% of the total additive genetic variance for rectal temperature, respiration rate score, and drooling score in Holstein cattle

Trait	Chromosome	Regions, Mb	Genetic variance explained, %	Candidate genes
Rectal temperature	BTA3	27.97–28.20	0.21	*NGF,*
	BTA4	24.75–24.97	0.17	*CRPPA, SOSTDC1*
	BTA5	11.11–11.25	0.23	*PPFIA2*
	BTA6	52.82–53.08	0.2	
	BTA6	60.70–60.82	0.16	*TMEM33*
	BTA8	79.10–79.56	0.25	*NAA35, GOLM1, ISCA1, TUT7*
	BTA14	21.35–21.36	0.24	
	BTA14	73.81–74.05	0.15	*OSGIN2, RIPK2*
	BTA21	46.59–46.82	0.19	*NKX2–1, NKX2–8*
	BTA22	3.39–3.57	0.4	
	BTA24	58.68–58.86	0.22	*PMAIP1*
	BTA25	25.60–25.89	0.19	*XPO6, SBK1, LAT, SPNS1, NFATC2IP*
	BTA28	5.81–5.96	0.15	*NTPCR, PCNX2*
	BTA29	38.30–38.69	0.18	*PAG14, PAG16, PAG20, PAG21, PAG1, PAG19*
Respiration rate score	BTA1	11.73–11.92	0.2	
	BTA1	63.04–63.26	0.17	
	BTA2	11.79–11.95	0.16	
	BTA3	0.45–0.61	0.19	*TIPR, GPR161, DCAF6*
	BTA7	71.05–71.22	0.15	
	BTA8	68.94–69.08	0.18	*GFRA2*
	BTA8	71.00–71.19	0.16	
	BTA9	43.41–43.64	0.22	*RTN4IP1, CRYBG1*
	BTA9	100.64–100.81	0.18	*PDE10A*
	BTA12	20.93–21.07	0.16	*WDFY2*
	BTA12	21.84–22.08	0.21	*MRPS31, FOXO1*
	BTA14	21.59–21.61	0.37	
	BTA14	61.23–61.38	0.22	*BAALC*
	BTA15	26.67–26.84	0.28	
	BTA17	6.06–6.22	0.15	*GATB; FAM160A1*
	BTA23	1.11–1.50	0.2	
	BTA24	36.50–36.82	0.19	
	BTA29	4.88–5.08	0.18	*CHORDC1*
	BTA30	120.98–121.08	0.17	
Drooling score	BTA3	31.88–32.01	0.19	*OVGP1*
	BTA3	49.52–49.63	0.26	*ABCA4, GCLM, DNTTIP2*
	BTA3	51.62–51.85	0.16	*TGFBR3*
	BTA3	52.17–52.2	0.18	
	BTA3	53.14–53.3	0.18	*ZNF326*
	BTA3	55.66–56.01	0.16	
	BTA6	37.35–37.38	0.16	*NCAPG*
	BTA6	38.20–38.23	0.21	
	BTA11	34.30–34.71	0.17	
	BTA11	34.86–35.08	0.21	
	BTA12	65.62–65.83	0.18	*GPC5*
	BTA12	65.96–66.18	0.27	*GPC5*
	BTA13	49.30–49.48	0.16	
	BTA13	55.36–55.62	0.25	
	BTA14	25.58–25.73	0.15	
	BTA16	9.08–9.30	0.20	
	BTA16	69.97–70.17	0.25	
	BTA21	24.80–24.94	0.33	*HDGFL3, TM6SF1*
	BTA21	57.87–58.10	0.17	*BTBD7, UNC79*
	BTA24	47.70–47.91	0.17	*ZBTB7C*

Many windows with small effects regulated RT, RR, and DS co-occurred, indicating that these three physiological indicators of heat stress are highly polygenic traits. A total of 54 protein-coding genes (27, 14, and 13 protein-coding genes located by the genomic region associated with RT, RS, and DS, respectively) were annotated in these genomic regions according to the Ensembl database. The GO enrichment and KEGG pathway analyses of the 54 protein-coding genes for the three physiological traits revealed 68 significant GO terms (Additional file 1: Fig. S1) including biological process as well as molecular functions and two significant KEGG pathway (purine metabolism and thiamine metabolism).

### General features and genetic background of cows sampled for RNA sequencing analysis

**Fig. 2:**
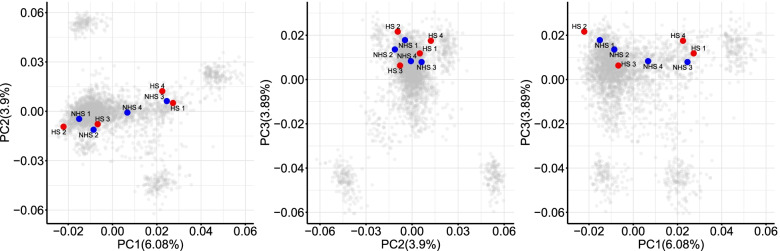
SNP-based principal component analysis of genotyped animals showing the diversity of animals selected for RNA sequencing samples. Red points represent the four heat stress season (HS) individuals, blue points represent the four non-heat stress season (NHS) individuals, and grey points represent the other individuals used in the weighted single-step genome-wide association study

The environmental THI and physiological performance (RT, RR, and DS) parameters during the two sample collection periods (April is the thermoneutral season in Beijing, named non-heat stress group, while July is the heat stress season, named heat stress group) were significantly different. The milk yield of the cows in the non-heat stress group (41.48 ± 3.52) was significantly higher than the cows in the heat stress group (35.25 ± 3.71). The description of environmental THI, physiological performance, and milk yield during periods of blood samples collecting are shown in Table 4. The genetic relationship of the dairy population evaluated is presented in Fig. 2, which shows a diverse genetic background of the cows (red and blue dots) sampled for the RNA-seq analysis.

**Table 4:** Summary statistics for environmental THI, physiological performance, and milk yield in April (non-heat stress) and July (heat stress)

Index at test day	Non-heat stress (April)	Heat stress (July)
Rectal temperature	38.50 ± 0.19	39.3 ± 0.67
Respiration rate score	37.67 ± 6.37	95.17 ± 16.30
Drooling score	1 ± 0.00	2 ± 0.82
Milk yield	41.48 ± 3.52	35.25 ± 3.71
THI	61.37 ± 8.18	81.62 ± 3.89

### Identification of differentially expressed mRNAs by sequencing

**Fig. 3:**
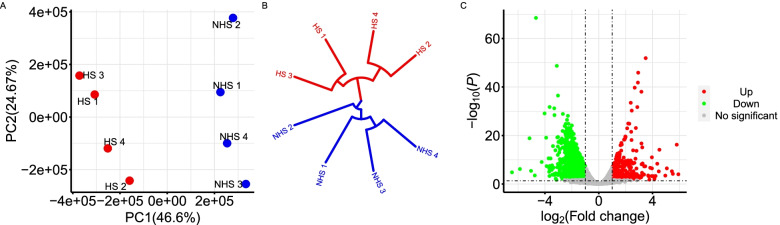
Cluster of heat stress and non-heat stress on the basis of read counts and volcano plot displaying differentially expressed genes. **A**, **B** Principle component analysis and dendrogram for samples collected under heat stress and non-heat stress on the basis of read counts. Red points in dot plot and red lines in dendrogram represent heat stress samples. Blue points in dot plot and blue lines in dendrogram represent heat stress samples. **C** Volcano plot showing significantly expressed genes. The red and green dots denote significantly downregulated and upregulated genes, respectively

**Fig. 4:**
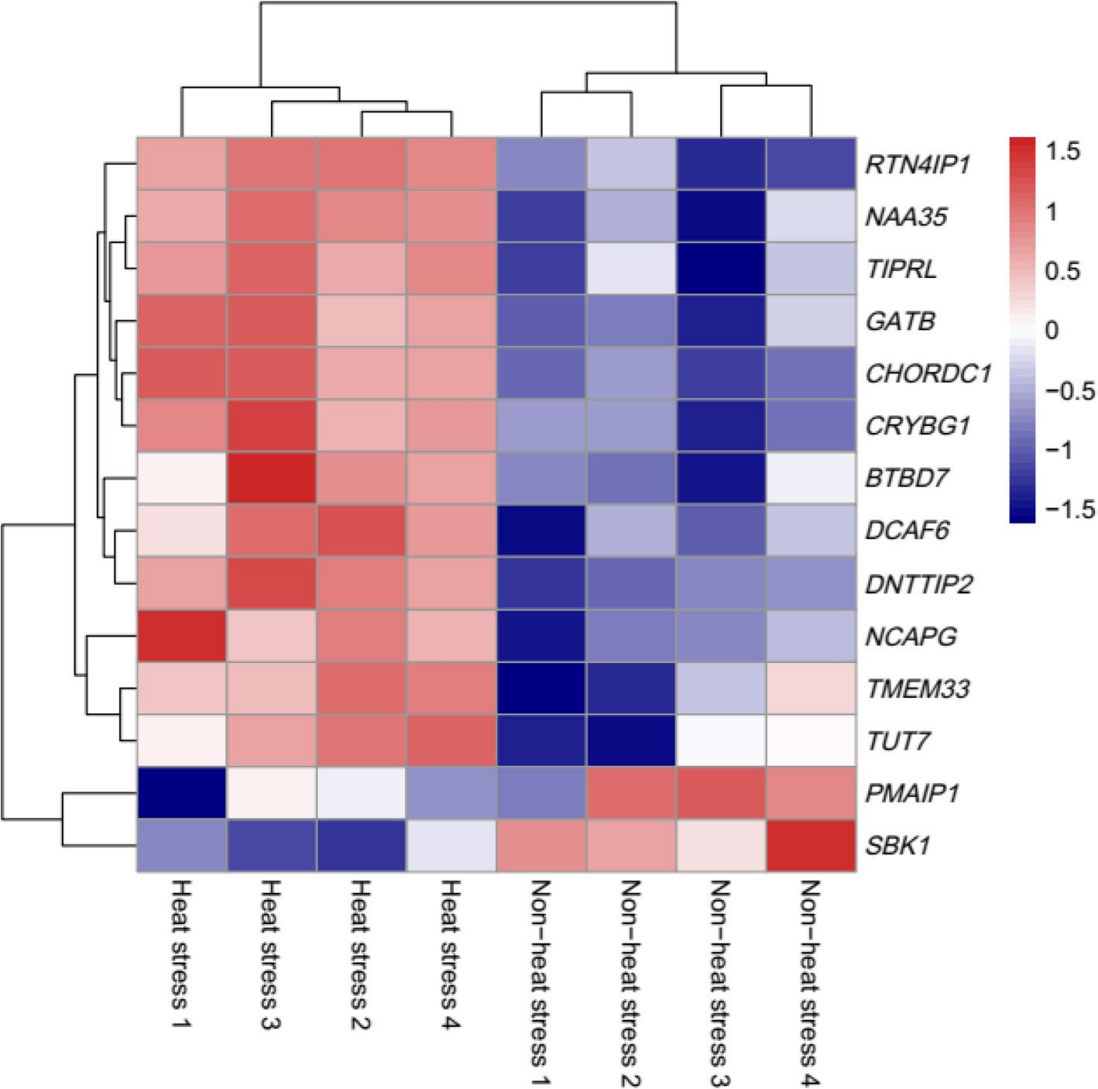
Heat map showing the relative normalized expression of the mRNA of 14 genes in eight individuals between period of April-non-heat stress and July-heat stress using hierarchical cluster. Genes with increased expression are shown in red while those with decreased expression are shown in blue

There is a significant difference between the heat stress (HS) group and non-heat stress (NHS) group for RNA-seq counts based on principle component analysis and clustering structure (Fig. 3). The analysis of the differently expressed genes (DEGs) were detected based on the quantile-adjusted conditional maximum likelihood method. A total of 2627 significantly downregulated genes were found and 369 upregulated genes in the NHS group compared to HS group (Additional file 2: Table S1). The GO enrichment and KEGG pathway analysis of DEGs revealed 21 significant GO terms including Biological Process, Cellular Component, and Molecular Function and 86 significant KEGG pathways (Additional file 3: Fig. S2). There were 14 genes identified based on the WssGWAS that were also DEGs. Table 5 shows that two of the significant common genes are upregulated and the other ones are downregulated. Figure 4 shows the heatmap of the mRNA expression of these 14 genes using hierarchical cluster of log2 from the relative normalized expression in bovine blood transcriptome.

**Table 5:** List of overlapping genes between weighted single-step genome-wide association analysis and RNA sequencing

Trait	Gene symbol	Log_2 _Ratio	FDR
		(Non-heat stress/ Heat stress)	
Rectal temperature	*PMAIP1*	1.21	1.11E-02
Rectal temperature	*SBK1*	1.73	1.41E-03
Rectal temperature	*TMEM33*	−1.35	4.35E-03
Rectal temperature	*TUT7*	−1.39	5.92E-03
Rectal temperature	*NAA35*	−1.09	3.47E-05
Respiration rate score	*GATB*	−1.36	2.44E-05
Respiration rate score	*CHORDC1*	−1.64	1.99E-10
Respiration rate score	*TIPRL*	−1.36	1.91E-04
Respiration rate score	*DCAF6*	−1.01	1.97E-04
Respiration rate score	*RTN4IP1*	−1.14	4.96E-05
Respiration rate score	*CRYBG1*	−1.14	6.40E-06
Drooling score	*BTBD7*	−1.02	1.19E-03
Drooling score	*DNTTIP2*	−1.37	1.48E-09
Drooling score	*NCAPG*	−1.53	7.59E-05

## Discussion

The estimation of genetic parameters and detection of genomic regions for three physiological indicators of heat stress in Holstein cattle contributes to further understanding of the genetic architecture of heat tolerance in cattle. The heritability estimates for the three traits are low (0.03–0.06) in the population evaluated but statistically different than zero. The variance component results utilizing the ***H*** matrix are similar to earlier estimates for the same traits using the numerator relationship matrix based on pedigree (***A*** matrix; [10], indicating that the size of the datasets used is large enough to accurately estimate genetic parameters for these three traits. The heritability estimated for RT in 1695 lactating Holstein cows during heat stress (0.17 ± 0.13; [37]) and in 3396 straight-bred and crossbred Romosinuano, Brahman, and Angus cattle (0.19 ± 0.03; [38] using bivariate models with coat score are higher than the estimates observed in our population. However, their accuracies of estimation are lower due to their smaller datasets.

For low-heritability traits, substantial genetic progress can be achieved by increasing intensity and accuracy of selection [39, 40]. Subsequently, genomic selection could significantly speed up the rates of genetic progress [41] by improving accuracy of selection and reducing generation interval. In our earlier study [12], single-SNP regression GWAS was performed for RT in a subset of the current population including 7598 Chinese Holstein cattle with 1114 genotyped cows. Ten SNPs (located on BTA3, BTA4, BTA8, BTA13, BTA14, and BTA29) were found to be significantly associated with RT and five positional candidate genes were identified (*SPAG17*, *FAM107B*, *TSNARE1*, *RALYL*, and *PHRF1*). In the present study we identified 53 trait-related genomic regions and 53 protein-coding genes through WssGWAS. Contrary to the single-SNP GWAS, more genomic regions were captured via WssGWAS (e.g., [42, 43], showing that WssGWAS can be more successful in detecting QTL. According to study of simulation [42], single-step GWAS (based on single-step genomic best linear unbiased prediction) could take care of correction for population structure in GWAS like other mixed linear models (e.g. EMMAX). Comparing to the method of WssGWAS, BayesB appears to overly shrink regions to zero, while overestimating the amount of genetic variation attributed to the remaining SNP effects in chicken population [24]. We compared two scenarios (weighting step including re-estimated GEBV and weighting step only with re-calculated SNP effect [24]) along with the inclusion of times of iteration for weighting (up to 5 iterations, results not shown). Although, the process of WssGWAS with only re-calculated SNP effects or more than three iterations resulted in too much noise (results not shown), which is consistent with prior studies based on simulated data in dairy cattle [44] and real data in broiler chickens [24]. Additionally, it is critical to select the number of SNPs included in the sliding windows when performing (W)ssGWAS as wider windows could capture more genomic regions [45, 46]. The SNP chip used has an average inter-marker spacing of 26.07 kb, and the average distance between adjacent SNPs with linkage disequilibrium (r^2^) higher than 0.4 was 200 kb in the studied population. Therefore, we selected 10-SNP windows in linkage disequilibrium when scanning QTL related to the three physiological traits.

RNA-seq analysis contributes to annotating new genes and splice variants, and provides cell- and context-specific quantification of gene expression. Research over the last few years has identified some of the physiological, metabolic, cellular, and molecular responses to heat stress in cattle [47, 48]. However, the level of gene expression depends on the physiological state of the organism and tissue analyzed. Cellular and transcriptomic adaptation of bovine granulosa cells were characterized to different heat stress intensities (39 °C, 40 °C, and 41 °C) in-vitro [49]. Several heat-responsive genes from different functional classes were identified and their associated pathways related to heat stress chaperons, cell death, apoptosis, hormonal synthesis, and oxidative stress. The expression of miRNAs in dairy cattle mammary gland under heat stress was investigated [50] (483 known bovine miRNAs and 139 novel miRNAs were identified), which detected the heat-dependent differential modulation of miRNAs. Decreased expression of heat response-associated genes in blood during HS was also observed very recently in lactating cows [28]. However, it was not considered that if metabolism of the milking stage from Spring to Summer directly affects the gene expression levels. In the current study of RNA-seq, we selected primiparous cows with similar DIM to avoid the effect of different lactation stage and the identified DEGs involved in the pathway of apoptosis, cellular senescence and autophagy, known to be affected by heat stress.

Validation of the results of GWAS is an essential component of the experimental program. It could increase the level of trust held by animal breeders in genetic improvement, and is important as confirmation of a scientific hypotheses [51]. There are many methods used to verify identified candidate genes/QTLs from GWAS results, e.g., SNP chip analysis [52], qRT-PCR [12], or association analysis in another population [53]. RNA-seq with the capabilities of high-throughput sequencing, could help us better understand the translation of genetic loci into biological mechanisms that underlie phenotypic expression of important traits [54]. Fourteen genes identified in WssGWAS were significantly and differently expressed between cows in heat stress period and thermoneutral conditions. *PMAIP1* is a proapoptotic member of the BCL-2 protein family that acts as a proapoptotic sensitizer/de-repressor and regulates diverse cellular functions in autophagic cell death and metabolism [55, 56]. When comparing to heat shock of 38 °C and 43 °C, the expression of *PMAIP1* was significantly and differentially upregulated in control (32 °C) mouse spermatogenic cells [57]. Another upregulated gene during heat stress based on the differential gene analysis is *SBK1,* which has been reported as part of widespread expression pattern involved in the protection of cells from apoptosis induced by the viral infection in ovarian cancer cells and promoting the cellular survival [58].

Five genes (*TMEM33*, *GATB*, *CHORDC1*, *RTN4IP1*, and *BTBD7*), out of the 12 significantly downregulated genes, were identified in prior studies involving heat stress/heat shock and cellular adaptive functions in the presence of stressors or disrupting molecular mechanisms. For instance, the overexpression of *TMEM33* cause expression of endoplasmic reticulum stress-induced cell death signals increase, which leads to activation of the unfolded protein response signaling cascade and induction of an apoptotic cell death [59]. The gene *GATB* was identified to show significant transcriptional differences (downregulated gene) in response to heat shock where a temperature shift from 37 °C to 42 °C was accessed through the use of a microarray [60]. The *GATB* gene downregulation is shown to be responsible for the respiratory chain enzyme deficiencies [61], and thus disturbing the mitochondrial function and possesses complications for the much-needed energy intensive physiological mechanisms to dissipate incremental heat load in cattle. Furthermore, during the temperature increase phase, *GATB* composed an operon and was downregulated in *Clostridium botulinum* (temperature shift from 37 °C to 45 °C over a period of 15 min; [62]. GO annotations related to *CHORDC1* include Hsp90 protein binding, and this gene was associated with the regulation of heat stress and immune response processes in rats [63]. Additionally, upregulation of *CHORDC1* is shown to be primarily associated with dystrophic conditions [64]. Therefore, it can be assumed that the downregulation of *CHORDC1* is a positive adaptive sign of mechanisms involving thermotolerance. *RTN4IP1* (Reticulon 4 Interacting Protein 1) is included in the gene ontology of oxidoreductase activity and transferase activity. The proteins of *RTN4IP1* were down-regulated at 26 °C compared to 18 °C in wild-type zebrafish [65]. The expression of *RTN4IP1* tend to decrease in the presence of stressors and decreased HSPs response, which again points towards a sort of possible homeostatic cellular response mechanisms initiated by the heat stressed dairy cow. *BTBD7* [BTB (POZ) domain-containing 7] is involved in a variety of biological functions [66]. *BTBD7* has been shown to be induced by a matrix protein at sites of cleft progression and induce a transcription factor and suppress cell adhesion [67]. Other studies described *BTBD7* as a cell growth suppressor protein (*ZNF238* is expressed in postmitotic brain cells and inhibits brain tumor growth) and a promotor of angiogenesis [68]. This function points out to the possible conservation mechanisms of heat stressed cows showing high energetic mechanisms directed at attaining thermo-neutrality and mechanisms of vasodilation of peripheral blood distribution network [69]. Query of literature strongly support the involvement of the possible candidate genes proposed in this study, in the regulatory mechanisms directed at differential homoerotic and homeostatic mechanisms manifesting in various physiological modifications towards heat stress, as described in previous studies [70]. Given the relevant support from prior research studies about the possible involvement in heat stress/heat shock response, the identification of genomic regions and candidate genes through WssGWAS, and biological validation through RNA-seq analyses indicate seven genes (*PMAIP1*, *SBK1*, *TMEM33*, *GATB*, *CHORDC1*, *RTN4IP1*, *BTBD7*) as likely playing an important role in heat stress response. Further studies should investigate these candidate genes in more depth and in more controlled on-farm or in-vitro experiments in response to more divergent environmental heat loads.

## Conclusions

Physiological indicators of heat tolerance are heritable and can be improved through direct selection. We identified 54 candidate genes associated with rectal temperature, respiration rate score, and drooling score in Chinese Holstein cattle using WssGWAS. A validation experiment based on RNA-seq of blood samples in Holstein cattle provided evidence of their possible role in the physiological responses to heat stress. The identified candidate genes (*PMAIP1*, *SBK1*, *TMEM33*, *GATB*, *CHORDC1*, *RTN4IP1*, *BTBD7*) may provide knowledge for developing genomic evaluation models by assigning higher weights to genetic markers located in these regions or the development of SNP panels contained polymorphisms in these genes.

## Supplementary Information

**Additional file 1: Fig. S1**. The 68 significant GO terms revealed by 54 protein-coding genes associated with the three physiological traits.

**Additional file 2: Table S1**. Differential gene expression between non-heat stress group and heat stress group.

**Additional file 3: Fig. S2**. The significant GO terms and pathways were enriched by Differential gene expression between non-heat stress group and heat stress group.

## Data Availability

The data sets used during the current study are available from the corresponding author on reasonable request.

## Abbreviations

RT: Rectal temperature
RR: Respiration rate score
DS: Drooling score
QTL: Quantitative trait loci
GWAS: Genome-wide association studies
RNA-seq: RNA sequencing
NGS: Next generation sequencing
WssGWAS: Weighted single-step GWAS
THI: Temperature-humidity index
DIM: Days in milk
AI-REML: Average information-restricted maximum likelihood
KEGG: Kyoto Encyclopedia of Genes and Genomes
GO: Gene Ontology
DEGs: Differently expressed genes
HS: Heat stress
NHS: Non-heat stress season

## References

[1] Schär C, Vidale PL, Lüthi D, Frei C, Häberli C, Liniger MA, Appenzeller C (2004). The role of increasing temperature variability in European summer heatwaves. Nature..

[2] Das R, Sailo L, Verma N, Bharti P, Saikia J, Imtiwati KR (2016). Impact of heat stress on health and performance of dairy animals: a review. Vet World.

[3] Polsky L, von Keyserlingk M (2017). Invited review: effects of heat stress on dairy cattle welfare. J Dairy Sci.

[4] Sejian V, Bhatta R, Gaughan JB, Dunshea FR, Lacetera N (2018). Review: adaptation of animals to heat stress. Animal..

[5] Ju XH, Xu HJ, Yong YH, An LL, Jiao PR, Liao M (2014). Heat stress upregulation of toll-like receptors 2/4 and acute inflammatory cytokines in peripheral blood mononuclear cell (PBMC) of Bama miniature pigs: An in vivo and in vitro study. Animal..

[6] Jin Y, Hu Y, Han D, Wang M (2011). Chronic heat stress weakened the innate immunity and increased the virulence of highly pathogenic avian influenza virus H5N1 in mice. J Biomed Biotechnol.

[7] Bagath M, Krishnan G, Devaraj C, Rashamol VP, Pragna P, Lees AM, Sejian V (2019). The impact of heat stress on the immune system in dairy cattle: a review. Res Vet Sci.

[8] Sammad A, Wang YJ, Umer S, Lirong H, Khan I, Khan A (2020). Nutritional physiology and biochemistry of dairy cattle under the influence of heat stress: consequences and opportunities. Animals..

[9] Parkhurst AM (2010). Model for understanding thermal hysteresis during heat stress: a matter of direction. Int J Biometeorol.

[10] Luo H, Brito LF, Li X (2021). Genetic parameters for rectal temperature, respiration rate, and drooling score in Holstein cattle and their relationships with various fertility, production, body conformation, and health traits. J Dairy Sci.

[11] Chen Z, Brito LF, Luo H, Shi R, Chang Y, Liu L, et al. Genetic and genomic analyses of service sire effect on female reproductive traits in Holstein cattle. Front Genet. 2021;12:713575. 10.3389/fgene.2021.713575. 10.3389/fgene.2021.713575 PMC8446201 34539741

[12] Luo H, Li X, Hu L (2021). Genomic analyses and biological validation of candidate genes for rectal temperature as an indicator of heat stress in Holstein cattle. J Dairy Sci.

[13] Wang H, Misztal I, Aguilar I, Legarra A, Muir W (2012). Genome-wide association mapping including phenotypes from relatives without genotypes. Genet Res.

[14] Freitas PHF, Oliveira HR, Silva FF, Fleming A, Miglior F, Schenkel FS, Brito LF (2020). Genomic analyses for predicted Milk fatty acid composition throughout lactation in north American Holstein cattle. J Dairy Sci.

[15] Oliveira HR, Lourenco DAL, Masuda Y (2019). Single-step genome-wide association for longitudinal traits of Canadian Ayrshire, Holstein, and Jersey dairy cattle. J Dairy Sci.

[16] Guarini AR, Lourenco DAL, Brito LF, Sargolzaei M, Baes CF, Miglior F, Misztal I, Schenkel FS (2019). Genetics and genomics of reproductive disorders in Canadian Holstein cattle. J Dairy Sci.

[17] Shi R, Brito LF, Liu A (2021). Genotype-by-environment interaction in Holstein heifer fertility traits using single-step genomic reaction norm models. BMC Genomics.

[18] Zhao B, Luo H, Huang X, Wei C, Di J, Tian Y, et al. Integration of a single-step genome-wide association study with a multi-tissue transcriptome analysis provides novel insights into the genetic basis of wool and weight traits in sheep. Genet Sel Evol. 2021;53:56. 10.1186/s12711-021-00649-8. 10.1186/s12711-021-00649-8 PMC8247193 34193030

[19] Ozsolak F, Milos PM (2011). RNA sequencing: advances, challenges and opportunities. Nat Rev Genet.

[20] Browning BL, Zhou Y, Browning SR (2018). A one-penny imputed genome from next-generation reference panels. Am J Hum Genet.

[21] Aguilar I, Misztal I, Johnson DL, Legarra A, Tsuruta S, Lawlor TJ (2010). Hot topic: a unified approach to utilize phenotypic, full pedigree, and genomic information for genetic evaluation of Holstein final score. J Dairy Sci.

[22] VanRaden PM (2008). Efficient methods to compute genomic predictions. J Dairy Sci.

[23] Misztal I, Tsuruta S, Lourenco DAL, Masuda Y, Aguilar I, Legarra A, Vitezica Z (2018). Manual for BLUPF90 family programs.

[24] Wang H, Misztal I, Aguilar I (2014). Genome-wide association mapping including phenotypes from relatives without genotypes in a single-step (ssGWAS) for 6-week body weight in broiler chickens. Front Genet.

[25] Team RC. R: A (2021). Language and environment for statistical computing. R Foundation for statistical computing, Vienna, Austria.

[26] Durinck S, Spellman PT, Birney E, Huber W (2009). Mapping identifiers for the integration of genomic datasets with the R/Bioconductor package biomaRt. Nat Protoc.

[27] Yu G, Wang L, Han Y, He Q (2012). ClusterProfiler: An R package for comparing biological themes among gene clusters. OMICS: J Integr Biol.

[28] Liu G, Liao Y, Sun B, Guo Y, Deng M, Li Y, Liu D (2020). Effects of chronic heat stress on mRNA and miRNA expressions in dairy cows. Gene..

[29] Yue S, Wang Z, Wang L, Peng Q, Xue B. Transcriptome functional analysis of mammary gland of cows in heat stress and thermoneutral condition. Animals. 2020;10(6):1015. 10.3390/ani10061015 PMC7341491 32532099

[30] Rio DC, Ares MJ, Hannon GJ, Nilsen TW (2010). Purification of RNA using TRIzol (TRI reagent). Cold Spring Harb Protoc.

[31] Chen S, Zhou Y, Chen Y, Gu J (2018). Fastp: An ultra-fast all-in-one FASTQ preprocessor. Bioinformatics..

[32] Dobin A, Davis CA, Schlesinger F, Drenkow J, Zaleski C, Jha S, Batut P, Chaisson M, Gingeras TR (2013). STAR: ultrafast universal RNA-seq aligner. Bioinformatics..

[33] Picard. URL http://broadinstitute.github.io/picard/. Accessed 9 Dec 2020.

[34] Purcell S, Neale B, Todd-Brown K (2007). PLINK: a tool set for whole-genome association and population-based linkage analyses. Am J Hum Genet.

[35] McCarthy DJ, Chen Y, Smyth GK (2012). Differential expression analysis of multifactor RNA-Seq experiments with respect to biological variation. Nucleic Acids Res.

[36] Kolde R (2019). Pheatmap: pretty heatmaps.

[37] Dikmen S, Cole JB, Null DJ, Hansen PJ (2012). Heritability of rectal temperature and genetic correlations with production and reproduction traits in dairy cattle. J Dairy Sci.

[38] Riley DG, Chase CC, Coleman SW, Olson TA (2012). Genetic assessment of rectal temperature and coat score in Brahman, Angus, and Romosinuano crossbred and straightbred cows and calves under subtropical summer conditions. Livest Sci.

[39] Muranty H, Troggio M, Sadok IB, Rifaï MA, Auwerkerken A, Banchi E, et al. Accuracy and responses of genomic selection on key traits in apple breeding. Hortic Res-England. 2015;2:15060. 10.1038/hortres.2015.60. 10.1038/hortres.2015.60 PMC4688998 26744627

[40] Song H, Zhang J, Zhang Q, Ding X. Using different single-step strategies to improve the efficiency of genomic prediction on body measurement traits in pig. Front Genet. 2019;9:730. 10.3389/fgene.2018.00730. 10.3389/fgene.2018.00730 PMC6340005 30693018

[41] Wiggans GR, Cooper TA, VanRaden PM, Cole JB (2011). Technical note: adjustment of traditional cow evaluations to improve accuracy of genomic predictions. J Dairy Sci.

[42] Mancin E, Lourenco D, Bermann M, Mantovani R, Misztal I. Accounting for population structure and phenotypes from relatives in association mapping for farm animals: a simulation study. Front Genet. 2021;12:642065. 10.3389/fgene.2021.642065. 10.3389/fgene.2021.642065 PMC8117227 33995481

[43] Marques DBD, Bastiaansen JWM, Broekhuijse MLWJ, Lopes MS, Knol EF, Harlizius B, et al. Weighted single-step GWAS and gene network analysis reveal new candidate genes for semen traits in pigs. Genet Sel Evol. 2018;50:40. 10.1186/s12711-018-0412-z. 10.1186/s12711-018-0412-z PMC6080523 30081822

[44] Lourenco DAL, Fragomeni BO, Bradford HL, Menezes IR, Ferraz JBS, Aguilar I, Tsuruta S, Misztal I (2017). Implications of SNP weighting on single-step genomic predictions for different reference population sizes. J Anim Breed Genet.

[45] Cáceres P, Barría A, Christensen KA, Bassini LN, Correa K, Garcia B, et al. Genome-scale comparative analysis for host resistance against sea lice between Atlantic Salmon and Rainbow trout. Sci Rep-UK. 2021;11:13231. 10.1038/s41598-021-92425-3. 10.1038/s41598-021-92425-3 PMC8225872 34168167

[46] Dikmen S, Cole JB, Null DJ, Hansen PJ (2013). Genome-wide association mapping for identification of quantitative trait loci for rectal temperature during heat stress in Holstein cattle. PLoS One.

[47] Srikanth K, Kwon A, Lee E, Chung H (2017). Characterization of genes and pathways that respond to heat stress in Holstein calves through transcriptome analysis. Cell Stress Chaperones.

[48] Sengar GS, Deb R, Singh U (2018). Identification of differentially expressed microRNAs in Sahiwal (Bos Indicus) breed of cattle during thermal stress. Cell Stress Chaperones.

[49] Khan A, Dou J, Wang Y, Jiang X, Khan MZ, Luo H, et al. Evaluation of heat stress effects on cellular and transcriptional adaptation of bovine granulosa cells. J Anim Sci Biotechno. 2020;11:25. 10.1186/s40104-019-0408-8. 10.1186/s40104-019-0408-8 PMC7027041 32095238

[50] Li Q, Yang C, Du J, Zhang B, He Y, Hu Q, et al. Characterization of miRNA profiles in the mammary tissue of dairy cattle in response to heat stress. BMC Genomics. 2018;19:975. 10.1186/s12864-018-5298-1. 10.1186/s12864-018-5298-1 PMC6309072 30593264

[51] Wen H, Luo H, Yang M (2021). Genetic parameters and weighted single-step genome-wide association study for supernumerary teats in Holstein cattle. J Dairy Sci.

[52] Zhao B, Fu X, Tian K (2019). Identification of SNPs and expression patterns of FZD3 gene and its effect on wool traits in Chinese merino sheep (Xinjiang type). J Integr Agric.

[53] Dikmen S, Wang XZ, Ortega MS, Cole JB, Null DJ, Hansen PJ (2015). Single nucleotide polymorphisms associated with thermoregulation in lactating dairy cows exposed to heat stress. J Anim Breed Genet.

[54] Zhao B, Luo H, He J, Huang X, Chen S, Fu X, et al. Comprehensive transcriptome and Methylome analysis delineates the biological basis of hair follicle development and wool-related traits in merino sheep. BMC Biol. 2021;19:197. 10.1186/s12915-021-01127-9. 10.1186/s12915-021-01127-9 PMC8427949 34503498

[55] Elgendy M, Sheridan C, Brumatti G, Martin SJ (2011). Oncogenic ras-induced expression of Noxa and Beclin-1 promotes autophagic cell death and limits clonogenic survival. Mol Cell.

[56] Lowman XH, McDonnell MA, Kosloske A, Odumade OA, Jenness C, Karim CB, Jemmerson R, Kelekar A (2010). The proapoptotic function of Noxa in human leukemia cells is regulated by the kinase Cdk5 and by glucose. Mol Cell.

[57] Janus P, Toma-Jonik A, Vydra N (2020). Pro-death signaling of cytoprotective heat shock factor 1: upregulation of NOXA leading to apoptosis in heat-sensitive cells. Cell Death Differ.

[58] Wang P, Guo J, Wang F, Shi T, Ma D (2011). Human SBK1 is dysregulated in multiple cancers and promotes survival of ovary cancer SK-OV-3 cells. Mol Biol Rep.

[59] Sakabe I, Hu R, Jin L, Clarke R, Kasid UN (2015). TMEM33: a new stress-inducible endoplasmic reticulum transmembrane protein and modulator of the unfolded protein response signaling. Breast Cancer Res TR.

[60] Madsen ML, Nettleton D, Thacker EL, Edwards R, Minion FC (2006). Transcriptional profiling of mycoplasma hyopneumoniae during heat shock using microarrays. Infect Immun.

[61] Friederich MW, Timal S, Powell CA (2018). Pathogenic variants in glutamyl-tRNAGln Amidotransferase subunits cause a lethal mitochondrial cardiomyopathy disorder. Nat Commun.

[62] Liang W, Bi Y, Wang H, Dong S, Li K, Li J (2013). Gene expression profiling of clostridium botulinum under heat shock stress. Biomed Res Int.

[63] Dou J, Khan A, Khan MZ, Mi S, Wang Y, Yu Y, Wang Y (2020). Heat stress impairs the physiological responses and regulates genes coding for extracellular exosomal proteins in rat. Genes-Basel..

[64] Signorelli M, Ebrahimpoor M, Veth O (2021). Peripheral blood transcriptome profiling enables monitoring disease progression in dystrophic mice and patients. EMBO Mol Med.

[65] Toni M, Angiulli E, Miccoli G (2019). Environmental temperature variation affects brain protein expression and cognitive abilities in adult zebrafish (danio Rerio): a proteomic and behavioural study. J Proteomics.

[66] Rouillard AD, Gundersen GW, Fernandez NF, Wang Z, Monteiro CD, McDermott MG, et al. The harmonizome: a collection of processed datasets gathered to serve and mine knowledge about genes and proteins. Database. 2016;2016:baw100. 10.1093/database/baw100. 10.1093/database/baw100 PMC4930834 27374120

[67] Sakai T (2016). Development and regeneration of salivary gland toward for clinical application. Oral Sci Int.

[68] Tao YM, Huang JL, Zeng S (2013). BTB/POZ domain-containing protein 7: epithelial-mesenchymal transition promoter and prognostic biomarker of hepatocellular carcinoma. Hepatology (Baltimore, Md).

[69] Abbas Z, Sammad A, Hu L, Fang H, Xu Q, Wang Y. Glucose metabolism and dynamics of facilitative glucose transporters (GLUTs) under the influence of heat stress in dairy cattle. Metabolites. 2020;10(8):312. 10.3390/metabo10080312. 10.3390/metabo10080312 PMC7465303 32751848

[70] Collier RJ, Baumgard LH, Zimbelman RB, Xiao Y (2019). Heat stress: physiology of acclimation and adaptation. Anim Front.

