# Supplementary figures and images for: Weighted single-step GWAS and RNA sequencing reveals key candidate genes associated with physiological indicators of heat stress in Holstein cattle
Source: J Anim Sci Biotechnol. 2022 Aug 20;13:108. doi: 10.1186/s40104-022-00748-6 (PMC9392250; doi:10.1186/s40104-022-00748-6)

## The Most Enriched GO Terms

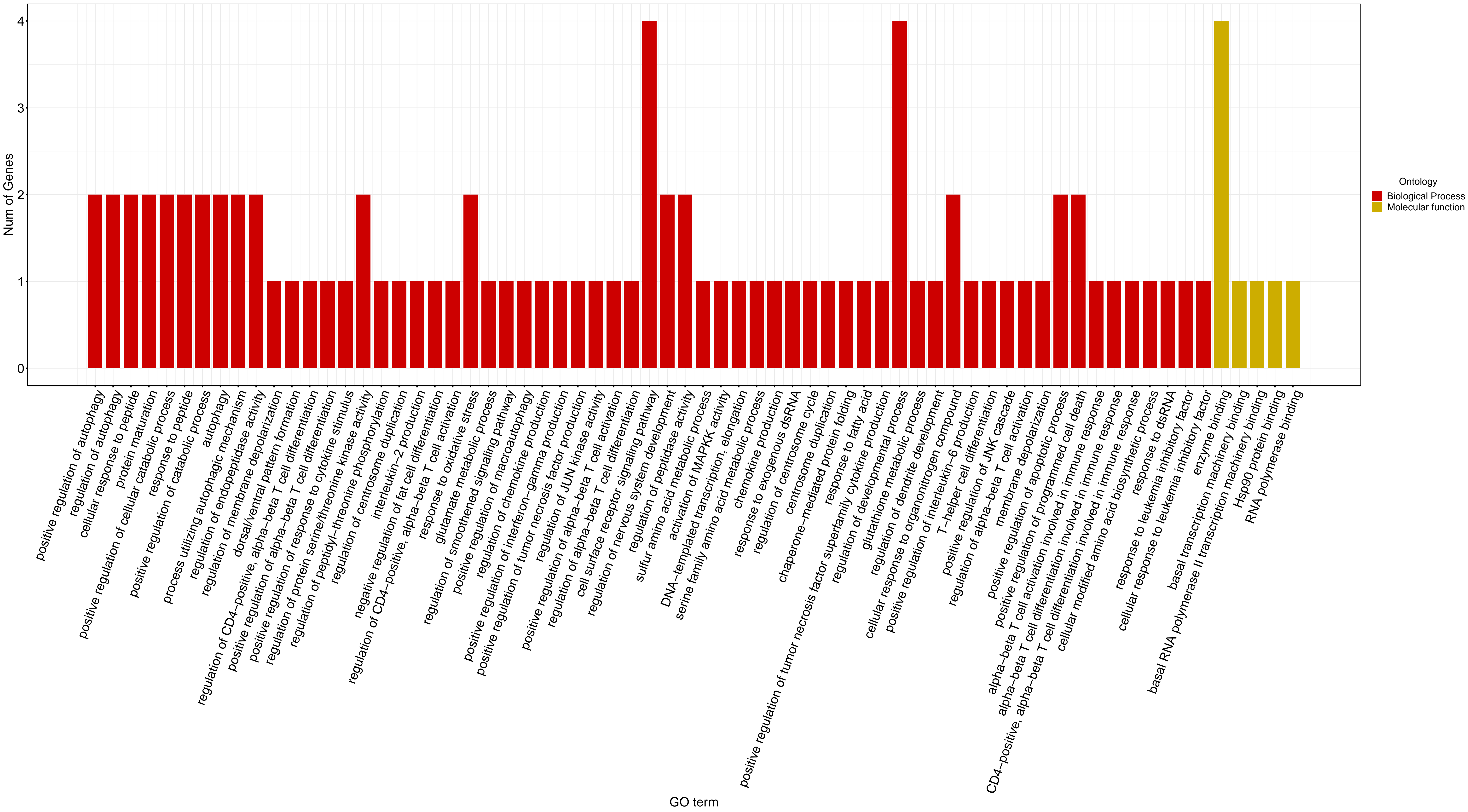

Supplement: Supplementary file 1 — Additional file 1: Fig. S1. The 68 significant GO terms revealed by 54 protein-coding genes associated with the three physiological traits. [file 40104_2022_748_MOESM1_ESM.pdf]

The Most Enriched GO Terms

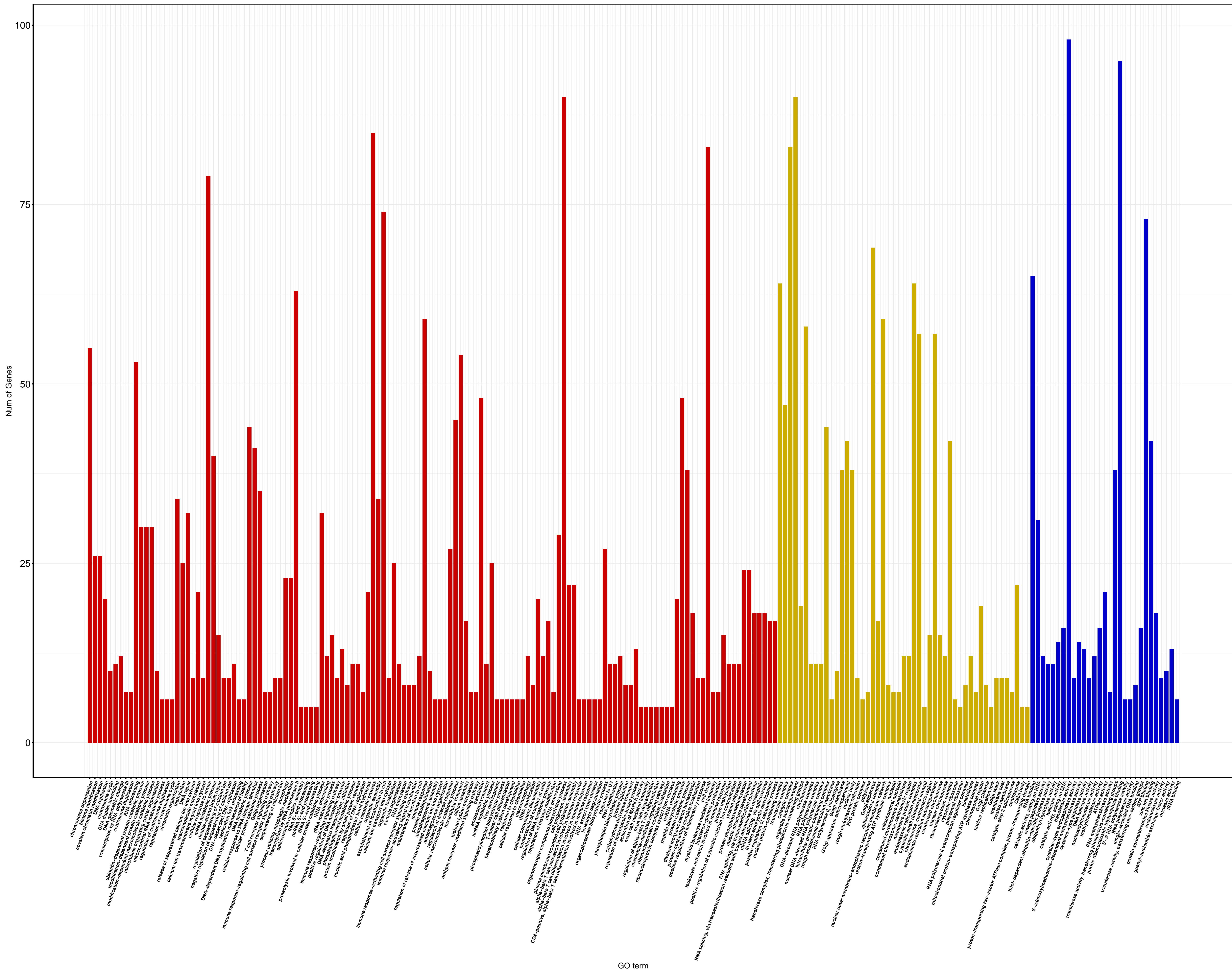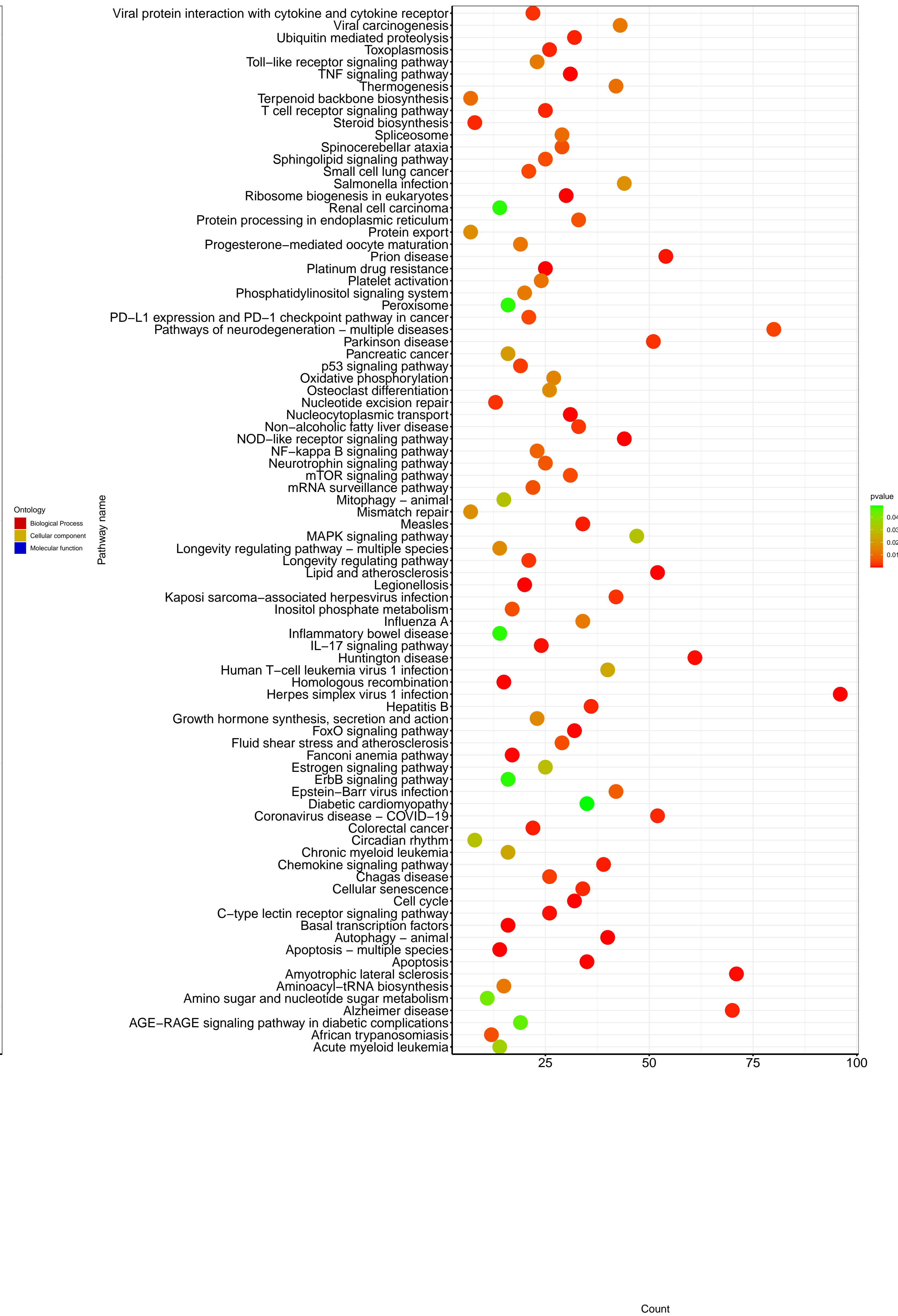

Supplement: Supplementary file 3 — Additional file 3: Fig. S2. The significant GO terms and pathways were enriched by Differential gene expression between non-heat stress group and heat stress group. [file 40104_2022_748_MOESM3_ESM.pdf]
